# A Case of Metastatic Ampulla of Vater Carcinoma Masked Under Recurrent Acute Pancreatitis: A Diagnostic Challenge

**DOI:** 10.7759/cureus.48675

**Published:** 2023-11-11

**Authors:** Ateeb Ur Rahman, Javeria Naveed, Muhammad Shoaib Asghar, Ahmed Ayan Asghar, Khadeja Azeem, Amna Chaudary, Munim Tariq

**Affiliations:** 1 Internal Medicine, Penn State Health, Camp Hill, USA; 2 Internal Medicine, Rashid Latif Medical College, Lahore, PAK; 3 Internal Medicine, The Rotherham NHS Foundation Trust, Sheffield, GBR

**Keywords:** ampulla of vater, metastatic adenocarcinoma of the ampulla of vater, adenocarcinoma, gastrointestinal malignancies, recurrent acute pancreatitis

## Abstract

The ampulla of Vater is a small opening located at the point where bile and pancreatic ducts join and empty their secretions into the small intestine. Ampullary cancers are rare but aggressive malignancies that can present with symptoms similar to those of acute pancreatitis, including abdominal pain, nausea, vomiting, and obstructive jaundice. Clinicians must rely on a combination of blood tests, imaging, and biopsies to diagnose ampullary cancer, which may be a hidden cause of acute pancreatitis. In this report, we present the case of a 66-year-old female who presented to our hospital with recurrent admissions due to abdominal pain, nausea, and vomiting. The patient was found to have repeated episodes of acute pancreatitis and was later diagnosed with cancer of the ampulla of Vater. This case proved extremely complex and diagnostically challenging.

## Introduction

The ampulla of Vater is a small opening near the confluence of the pancreatic and common bile ducts, and cancers that arise at this location are known as ampullary cancers [1]. Primary ampullary cancer is rare. Ampullary cancers are more common in patients with hereditary polyposis syndromes, such as familial adenomatous polyposis (FAP) and hereditary nonpolyposis colorectal cancer (HNPCC, Lynch syndrome) than in the general population [2]. According to the morphology and location, the ampulla of Vater cancers can be divided into three different categories: intra-ampullary neoplasms, periampullary neoplasms, and mixed neoplasms [3]. Occasionally, malignancy at the ampulla of Vater may cause recurrent episodes of pancreatitis [4].

Risk factors for ampulla of Vater carcinoma include a history of smoking, chronic pancreatitis, and a family history of gastrointestinal malignancies. Additionally, patient age can be a determining factor as ampullary tumors tend to occur in older individuals.

## Case presentation

A 66-year-old female, with a medical history of GERD, asthma, and morbid obesity, presented to the hospital with nausea, vomiting, abdominal pain, and diarrhea. The review of the systems was negative for any other symptoms, and physical examination findings were significant for diffuse abdominal pain. The patient had a history of a cholecystectomy. She denied any history of alcohol consumption. Laboratory tests showed elevated lipase at 1650, and abdominal CT showed inflammation of the body and tail of the pancreas, suggesting acute pancreatitis. Supportive management for pancreatitis was initiated. Symptoms improved within a few days. The patient was diagnosed with idiopathic pancreatitis and discharged on analgesics with outpatient gastroenterology follow-up.

The patient remained asymptomatic for one year until she was re-examined by a primary care physician for abdominal pain. Her symptoms were thought to be due to GERD and was started on a PPI trial with follow-up. A few weeks later, the patient was examined by the primary care physician for itching and was found to have scleral/palmar icterus on physical exam. Laboratory work showed elevated bilirubin up to 15 mg/dL and lipase up to 1500U/L. The patient was sent to the ER and admitted for acute recurrent pancreatitis. During hospitalization, the patient underwent an ultrasound of the liver and showed a CBD dilation of 14 mm with mild intrahepatic ductal dilation and moderate dilation of the pancreatic duct to 8 mm. Non-contrast MRCP was performed, which showed intra-and extrahepatic biliary ductal dilation, as well as dilation of the pancreatic duct throughout its course, which was not present on the prior CT examination. T2 hypointense focus of the distal aspect of the CBD at the confluence of the CBD and major pancreatic duct measured approximately 1.4 x 1 cm, most likely representing an obstructing calculus. However, an intraductal mass was not excluded in the non-contrast imaging study. No obvious pancreatic mass was found. The gastroenterology team was consulted. Endoscopic retrograde cholangiopancreatography (ERCP) was performed, pancreaticobiliary stents were placed, and biliary brushings were sent for cytology.

**Figure 1:**
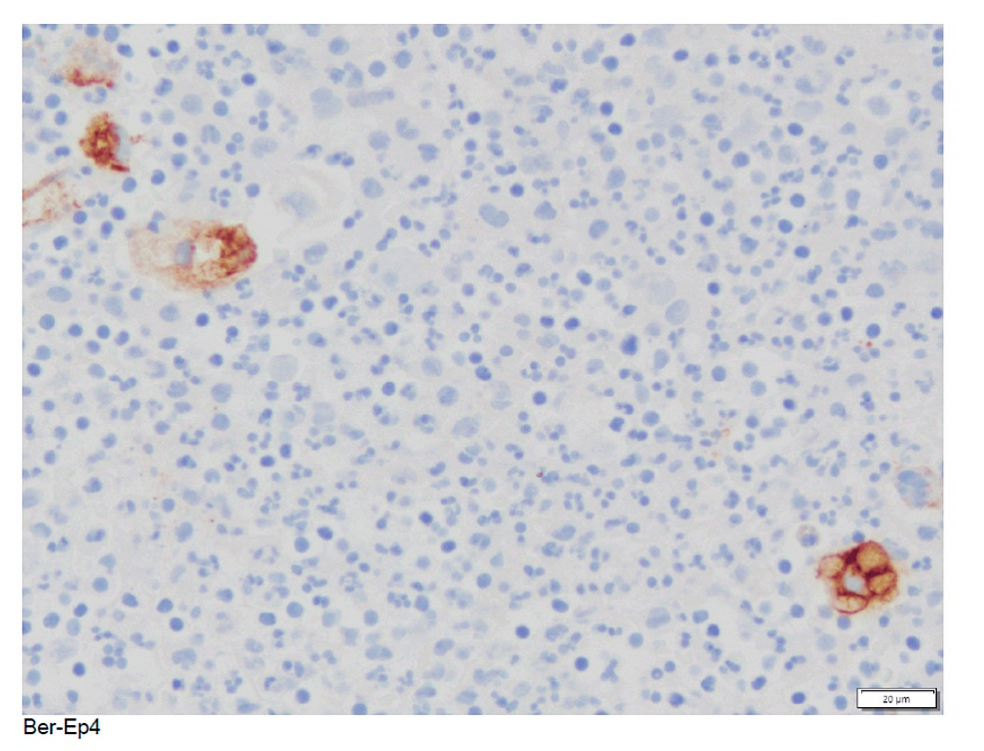
Ber-Ep4 – a monoclonal antibody that is found positive in epithelial-glycoprotein-adhesion-molecules (EpCAM), usually found on basal cell carcinoma (BCC) cells. This can otherwise be positive in most epithelial cells of the body.

The patient’s cytology was suboptimal due to extreme cellularity, a single group of atypical cells, and limited interpretation. The patient symptomatically improved and was scheduled to undergo repeat ERCP. The patient underwent repeat ERCP with cholangioscopy approximately one month after discharge. Prior biliary and pancreatic stents were removed, a bile duct stricture was identified, and biliary sphincterotomy with a stent was performed. Tissue pathology showed benign bile duct epithelium and subepithelial tissue with acute inflammation and focal fibrosis. A few cells of Ber-Ep4 (Figure 1) and GATA 3 (GATA binding protein 3) were found to be positive.

The patient was admitted to the hospital several times over the next few months because of recurrent acute pancreatitis. She underwent laboratory and imaging studies with similar findings of pancreatitis as before, and the patient was discharged home with outpatient follow-up. The patient was referred to a pancreatic specialist at a tertiary care center.

**Figure 2:**
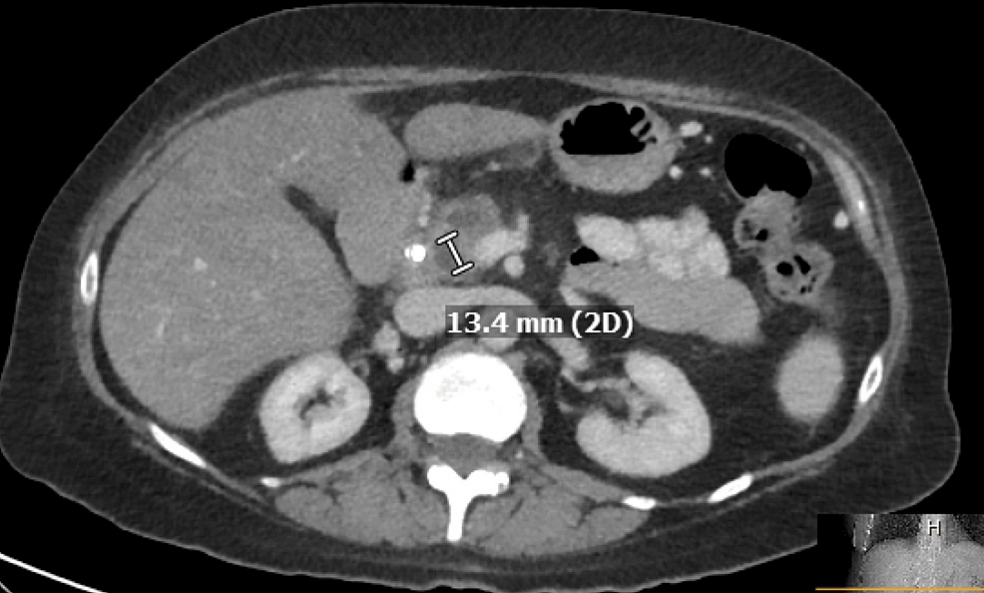
Mass at the ampulla of Vater is 13.4 mm in size.

**Figure 3:**
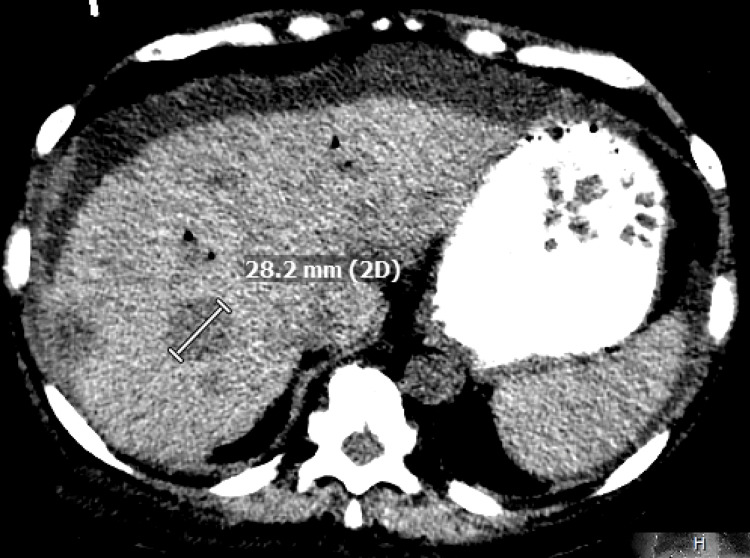
Multiple hypoechoic lesions of the liver. The largest one seen is 28.2 mm.

Unfortunately, the patient had another episode of pancreatitis and was admitted to the hospital. CT abdomen/pelvis was performed, which showed an enlarged and edematous pancreatic head, a possible mass at the ampulla of Vater of 13.4 mm (Figure 2), and multiple indeterminate hypoechoic masses of the liver (Figure 3).

Gastroenterology was consulted, and the patient underwent repeat ERCP. The biliary stricture was redilated, a stent was placed, and a biopsy was taken from the papilla. Pathology results were suggestive of metastatic ampullary/periampullary adenocarcinoma of the ampulla of Vater. The patient also had elevated CEA levels (150 ng/mL). Surgical oncology was consulted, but the patient was not found to be a suitable candidate. Hematology and oncology were consulted, and the patient was started on FOLFIRINOX chemotherapy. The patient received six cycles of chemotherapy, but due to the progression of the disease and clinical frailty, the family decided to opt for hospice care, and the patient passed away within a few months.

## Discussion

The ampulla of Vater is formed primarily by the union of the terminal intraduodenal portions of the common bile duct and main pancreatic duct, along with a small portion of the duodenal mucosa [1]. The ampulla of Vater is absent in one-third of the population, and in these individuals, the ducts open separately into the duodenum [1].

Carcinomas of the ampulla of Vater account for 0.2% of all gastrointestinal malignancies and 6% of all periampullary tumors and rarely occur in clinical practice compared to those from the common bile duct or pancreas. Hence, they are challenging to diagnose clinically, especially if the disease is masked by the underlying pathology of the pancreas or duodenum [2]. The rarity of the incidence and heterogeneous nature of the presentation of this cancer make it diagnostically and therapeutically challenging. Patients may present with symptoms that are routinely observed in extrahepatic cholangiocarcinoma, pancreatitis, or pancreatic carcinoma [3]. The patients present with apparently not unusual symptoms, including jaundice, diarrhea, steatorrhea, etc., that makes it challenging to have a suspicion about the ampulla of Vater carcinoma. The presentation of any patient with symptoms related to recurrent pathologies of the pancreas, bile duct, or duodenum pertains to the clinician with ampullary carcinoma in their differential diagnosis, which enables earlier diagnostic workup and leads to a higher chance of cure. Endoscopic ultrasound, endoscopic retrograde cholangiopancreatography, and fine needle aspiration cytology are widely regarded as the optimal diagnostic modalities for accurate and effective medical diagnosis [3].

This case report raises awareness about the limitations of imaging and biopsies in establishing a definitive diagnosis, especially when dealing with suboptimal samples or focal lesions [4]. In cases with persistent clinical suspicion despite initial negative results, repeat investigations or alternative diagnostic modalities should be considered [5]. Additionally, the importance of the double duct sign on imaging can raise the suspicion of a possible malignancy of the pancreatic head and ampulla of Vater [6].

ERCP is one of the most commonly used diagnostic procedures for evaluating the bile and pancreatic ducts. However, this procedure can be complicated by pancreatitis, which can lead to inflammation and edema of the pancreas, making it difficult to visualize the ampulla of Vater. MRCP and endoscopic ultrasound (EUS) are alternative imaging techniques that can help identify ampullary tumors and differentiate them from pancreatitis.

The standard surgical treatment is pancreaticoduodenectomy in cases where resection is feasible [7]. However, the involvement of the pancreaticoduodenal and para-aortic lymph nodes must be considered [5]. Endoscopic ampullectomy is a broadly accepted approach due to the minimum procedural trauma but has a limitation of the lack of lymphadenectomy. Hence, it could not be done on the patient since the patient presented with metastasis. Another treatment option is surgical ampullectomy, but it is much less preferred due to its plethora of complications [8]. Chemotherapy plays a vital role in effectively treating ampullary carcinoma, particularly in patients who present with distant metastasis, recurrent disease, or locally advanced disease that cannot be surgically removed [9].

It is also important to note a potential genetic predisposition associated with the ampulla of Vater carcinoma, as the patient in this case report had a family history of various cancers, including lung, kidney, and breast cancers. Therefore, genetic counseling and testing are recommended to evaluate the presence of any underlying genetic mutations. The patients, sometimes, have underlying mutations, such as BRCA, ATM, RAD50, and MUTYH [10], which may impact treatment decisions, including the use of PARP inhibitors [11]. PARP inhibitors (PARPi) such as Olaparib have been created to enhance the cytotoxicity of particular chemotherapeutic drugs and are presently being examined in combination with chemotherapy for a wide range of cancer types [12].

## Conclusions

This case report highlights the diagnostic challenges encountered in identifying metastatic ampulla of Vater carcinoma, emphasizing the limitations of imaging and biopsy, especially in cases with suboptimal samples or focal lesions. Persistent clinical suspicion, even in the face of initial negative results, should prompt the consideration of repeat investigations or alternative diagnostic modalities. These findings emphasize the critical need for refined diagnostic approaches and a high index of suspicion in similar cases to ensure timely and accurate diagnosis. Genetic counseling is vital for patients with a family history of cancer as it can significantly affect treatment decisions. Further research is essential to enhance our understanding of the optimal diagnostic and treatment strategies for metastatic ampulla of Vater carcinoma.

## References

[1] Paluri RK, Kasi A (2022). Ampullary cancer. StatPearls.

[2] Ahn DH, Bekaii-Saab T (2014). Ampullary cancer: an overview. Am Soc Clin Oncol Educ Book.

[3] Rizzo A, Dadduzio V, Lombardi L, Ricci AD, Gadaleta-Caldarola G (2021). Ampullary carcinoma: an overview of a rare entity and discussion of current and future therapeutic challenges. Curr Oncol.

[4] Gaspar B, Beuran M, Paun S, Ganescu R, Hostiuc S, Negoi I (2013). Current strategies in the therapeutic approach for adenocarcinoma of the ampulla of Vater. J Med Life.

[5] Fernandez-Cruz L (2001). Periampullary carcinoma. Surgical Treatment: Evidence-Based and Problem-Oriented.

[6] Agrawal S, Vohra S (2017). Simultaneous Courvoisier's and double duct signs. World J Gastrointest Endosc.

[7] Taliente F, Bianco G, Moschetta G, Franco A, Giovinazzo F, Agnes S, Spoletini G (2022). From endoscopic resection to pancreatoduodenectomy: a narrative review of treatment modalities for the tumors of the ampulla of Vater. Chin Clin Oncol.

[8] Ceppa EP, Burbridge RA, Rialon KL (2013). Endoscopic versus surgical ampullectomy: an algorithm to treat disease of the ampulla of Vater. Ann Surg.

[9] Regalla DK, Jacob R, Manne A, Paluri RK (2019). Therapeutic options for ampullary carcinomas. A review. Oncol Rev.

[10] Wong W, Lowery MA, Berger MF (2019). Ampullary cancer: evaluation of somatic and germline genetic alterations and association with clinical outcomes. Cancer.

[11] Mauri G, Gori V, Patelli G (2023). Multimodal treatment with curative intent in a germline BRCA2 mutant metastatic ampullary adenocarcinoma: a case report. World J Surg Oncol.

[12] Javle M, Curtin NJ (2011). The role of PARP in DNA repair and its therapeutic exploitation. Br J Cancer.

